# Responses of Arbuscular Mycorrhizal Symbiosis to Abiotic Stress: A Lipid-Centric Perspective

**DOI:** 10.3389/fpls.2020.578919

**Published:** 2020-11-12

**Authors:** Zengwei Feng, Xiaodi Liu, Honghui Zhu, Qing Yao

**Affiliations:** ^1^ College of Horticulture, Guangdong Province Key Laboratory of Microbial Signals and Disease Control, South China Agricultural University, Guangzhou, China; ^2^ State Key Laboratory of Applied Microbiology Southern China, Guangdong Provincial Key Laboratory of Microbial Culture Collection and Application, Guangdong Open Laboratory of Applied Microbiology, Guangdong Microbial Culture Collection Center (GDMCC), Guangdong Institute of Microbiology, Guangdong Academy of Sciences, Guangzhou, China

**Keywords:** abscisic acid, arbuscular mycorrhizae, abiotic stress, lipid metabolism, neutral lipids, phospholipids

## Abstract

Arbuscular mycorrhizal (AM) fungi are one of the most important soil microbial resources that help host plants cope with various abiotic stresses. Although a tremendous number of studies have revealed the responses of AM fungi to abiotic stress and their beneficial effects transferred to host plants, little work has focused on the role of lipid metabolism in AM fungi under abiotic stress conditions. AM fungi contain a large amount of lipids in their biomass, including phospholipids (PLs) in their hyphal membranes and neutral lipids (NLs) in their storage structures (e.g., vesicles and spores). Recently, lipid transfer from plants to AM fungi has been suggested to be indispensable for the establishment of AM symbiosis, and extraradical hyphae are capable of directly taking up lipids from the environment. This experimental evidence highlights the importance of lipids in AM symbiosis. Moreover, abiotic stress reduces lipid transfer to AM fungi and promotes arbuscule collapse as well as the hydrolysis and conversion of PLs to NLs in collapsed arbuscules. Overall, this knowledge encourages us to rethink the responses of AM symbiosis to abiotic stress from a lipid-centric perspective. The present review provides current and comprehensive knowledge on lipid metabolism in AM fungi, especially in response to various abiotic stresses. A regulatory role of abscisic acid (ABA), which is considered a “stress hormone,” in lipid metabolism and in the resulting consequences is also proposed.

## Introduction

Arbuscular mycorrhizal (AM) fungi, which phylogenetically belong to the subphylum Glomeromycotina, can form mutualistic symbiotic associations with more than 80% of terrestrial plant species (Smith and Read, 2008; Spatafora et al., 2016). This extremely ancient (>450 million years) and coevolutionary relationship is considered the key factor in early plant colonization of land and has also been verified to be generally beneficial to both partners (Remy et al., 1994; Smith and Read, 2008). AM fungi are obligate biotrophic fungi that rely exclusively on carbon in the form of lipids and sugars from their host plants to sustain their growth, development, and function (Bago et al., 2003; Helber et al., 2011; Jiang et al., 2017; Luginbuehl et al., 2017). In return, AM fungi are capable of helping their host plants grow vigorously under a variety of abiotic stress conditions by mediating a series of complex signal communications and enhancing the exchange of multiple substances between partners, which leads to enhanced physiological-biochemical traits and increased uptake of nutrients and water (Bitterlich et al., 2018; Begum et al., 2019; Evelin et al., 2019).

Abiotic stresses are widespread in terrestrial ecosystems and are becoming increasingly severe because of dramatic changes in the global climate, environmental pollution, and excessive human activities during the past several decades. In agricultural ecosystems, numerous studies have highlighted that AM fungi are capable of improving the tolerance of their host plants to drought (Grümberg et al., 2015; Li et al., 2019b), salinity (Augé et al., 2014; Chandrasekaran et al., 2019), heavy metals (Wu et al., 2018; Zhang et al., 2018, 2019; Rask et al., 2019), low nutrient availability (Tanaka and Yano, 2005; Garcia and Zimmermann, 2014; Koegel et al., 2015; Chu et al., 2020), extreme temperature (heat and cold; Zhu et al., 2011; Chen et al., 2013; Cabral et al., 2016; Mathur et al., 2018), acidic soils (low pH; Huang et al., 2017a; Wang et al., 2017; Feng et al., 2020), aluminum (Al) toxicity (Seguela et al., 2016; Aguilera et al., 2018), and pollutants (As and polycyclic aromatic hydrocarbons; Aranda et al., 2013; Calonne et al., 2014) to varying degrees. The mechanisms underlying the improved tolerance afforded by AM fungi involve increased nutrient levels, optimized water balance, enhanced photosynthesis, and increased reactive oxygen species (ROS) scavenging activity in plants. In contrast, however, the responses of AM fungi to abiotic stresses are largely neglected, hindering our understanding of AM symbiosis under stress conditions. Recently, lipids have been suggested to be the indispensable carbon forms delivered from plant cells to AM fungi (Jiang et al., 2017; Luginbuehl et al., 2017), highlighting the central role of lipids in regulating AM symbiosis. More recently, Feng et al. (2020) demonstrated that decreased transfer of lipids from root cells to AM fungi contributed to the inhibition of colonization and functionality of AM fungi in response to low-pH stress. Therefore, now is the right time to evaluate the role of lipids in AM symbiosis under various abiotic stress conditions. To address this topic, we discuss the cytobiochemical changes in plants and physiological changes in AM fungi in response to abiotic stress and focus on lipid metabolism in symbiosis.

## Cytobiochemical Changes in Plants in Response to Abiotic Stress

The optimal growth status of plants requires precise cellular homeostasis achieved by a delicate balance between multiple pathways in various cellular compartments (Miller et al., 2010). This coordination may, however, be disrupted rapidly when plants are exposed to a series of abiotic stresses. As a consequence of adverse conditions, different cytobiochemical changes in plants emerge concomitantly. These changes mainly include ROS generation, membrane lipid peroxidation, and increases in abscisic acid (ABA), all of which exert dramatic and regulatory effects on AM symbiosis.

ROS generation is the most significant event in plant cells subjected to abiotic stress; this generation occurs in several main organelles, e.g., chloroplasts, mitochondria, and peroxisomes, as well as in the plasma membrane and apoplast. ROS at high doses (ROS bursts) are capable of causing oxidative damage to many biomacromolecules (e.g., membrane lipids, proteins, RNA, and DNA), which ultimately results in cellular damage and even death (Apel and Hirt, 2004; Miller et al., 2010; You and Chan, 2015). In addition, at low doses, ROS function as signaling molecules in the induction of pathogen resistance by AM fungi (Zhang et al., 2013; Zhu et al., 2015). The membrane structures of plant cells and subcellular organelles are mainly composed of lipids, e.g., polyunsaturated fatty acids, which are highly sensitive to ROS (Tsikas, 2017). Under abiotic stress conditions, excessive lipid peroxidation can alter the assembly, composition, structure, and dynamics (fluidity) of membranes, further leading to membrane damage. Polyunsaturated fatty acids, which are long-chain fatty acids with more than one double bond, are preferentially oxidized to the final form malondialdehyde, either by chemical reactions with ROS or by enzymatic reactions catalyzed by lipoxygenase in the lipid peroxidation process (Gaschler and Stockwell, 2017; Tsikas, 2017). In roots colonized by AM fungi, the plasma membrane encapsulating arbuscules is highly specialized and is referred to as the periarbuscular membrane. However, the lipid peroxidation of the periarbuscular membrane has not yet been explored, let alone that of the arbuscular membrane. ABA is commonly known as a “stress hormone” and plays a crucial role in the plant response to abiotic stress (Fujii et al., 2009; Kim et al., 2016; Huang et al., 2017b). Under weakly stressing conditions, elevated ABA induces a mild increase in ROS (Jiang and Zhang, 2002). Intriguingly, AM fungal colonization can also trigger an increase in ROS, which further induces localized and systemic resistance to pathogens (Zhu et al., 2015). However, long-term and severe stress conditions can result in ROS bursts and cell damage.

## Arbuscular Mycorrhizal Fungal Responses to Abiotic Stress

Similar to their host plants, AM fungi also undergo various changes to acclimate abiotic stress. The adaptation of AM fungi to stressed conditions is mainly reflected by several different aspects, such as colonization, arbuscule formation, spore germination, and sporulation. In AM fungi, the hyphal membrane is composed of phospholipids (PLs), while spores contain a large amount of neutral lipids (NLs; Olsson and Johansen, 2000). Consequently, any changes in these structures are linked to the dynamics of lipid metabolism.

**Figure 1:**
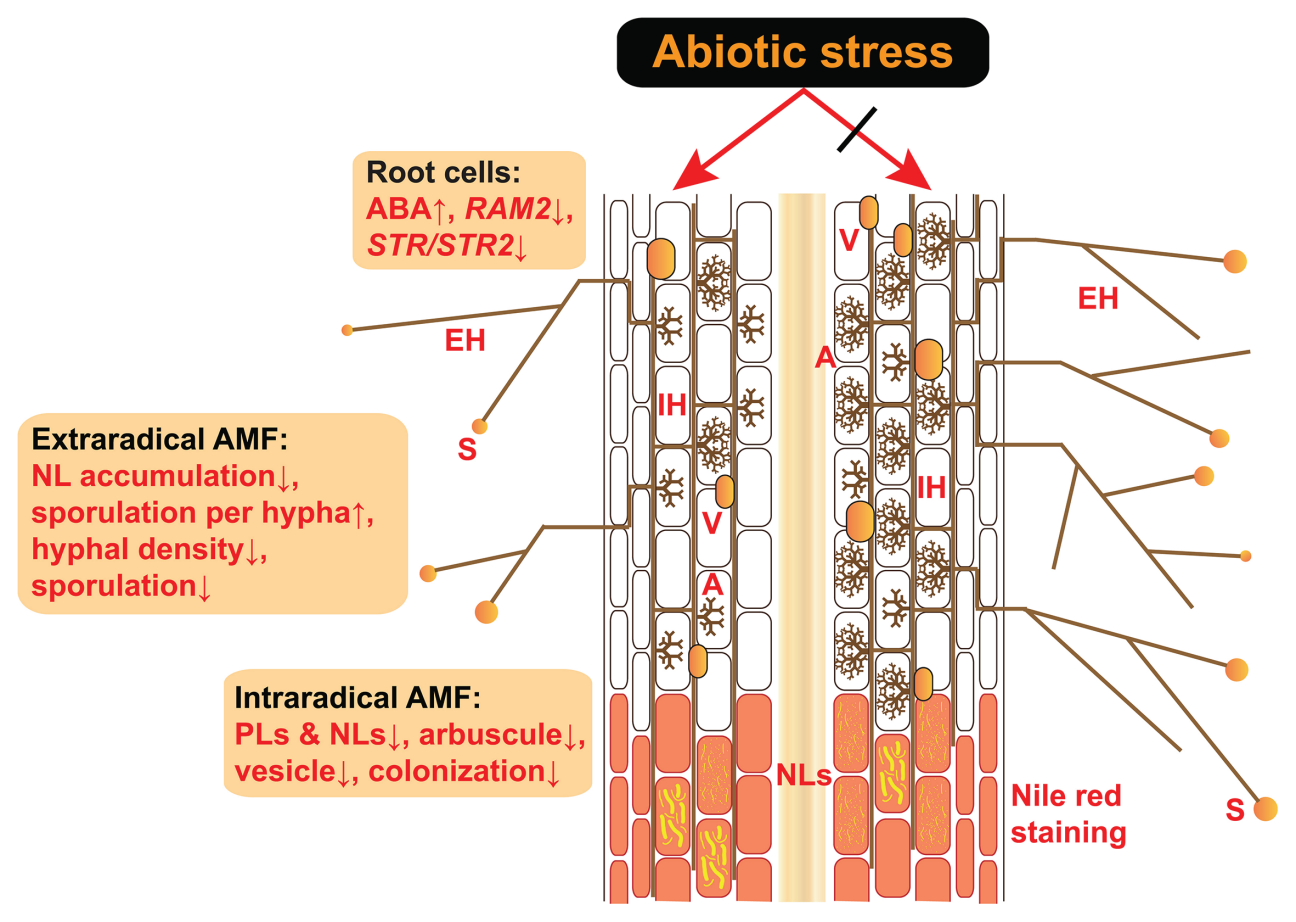
Effects of abiotic stress on the intraradical and extraradical structures of AM fungi and their host plant root cells. Abiotic stress induces an ABA increase in host plants as well as decreased expression of *RAM2* and *STR/STR2*, both of which are induced specifically by AM fungi and encode enzymes responsible for lipid biosynthesis and transfer from the roots to AM fungi (Bravo et al., 2017; Feng et al., 2020). Under abiotic stress, the accumulations of PLs and NLs are reduced in the intraradical phase, in parallel with decreased mycorrhizal colonization (e.g., arbuscule abundance, vesicle abundance, and colonization rate). In the extraradical phase, the accumulation of NLs in the hyphae and hyphal density is reduced in response to abiotic stress. Although the sporulation per hypha is accelerated by increased ABA (Liu et al., 2020), the total sporulation is inhibited due to the reduction in hyphal density. A, arbuscules; V, vesicles; IH, intraradical hyphae; EH, extraradical hyphae; S, spores; NLs, neutral lipids; PLs, phospholipids; ABA, abscisic acid; AM, arbuscular mycorrhiza.

Many studies have shown that abiotic stress has an overall negative effect on mycorrhizal colonization (Figure 1). Salinity-alkalinity stress significantly inhibits the quantities of entry points on roots and vesicles inside roots (Ye et al., 2019). Mycorrhizal frequency (F%) and intensity (M%) significantly decreases with increasing salinity (Krishnamoorthy et al., 2014). Drought, low temperature, and heavy metals were found to suppress mycorrhizal colonization (Chen et al., 2013; He et al., 2017; Zhang et al., 2019). Acidic soil decreased F% by 4.39% and M% by 20.30% in pot experiments (Liu et al., 2020), which is in accordance with previous results in axenic culture (Wang et al., 2017). Vesicle abundance at a pH of 4.5 was shown to be approximately half of that at a pH of 6.5 (Feng et al., 2020). Even so, Wang et al. (2017) indicated that extraradical hyphae (EH) of *Rhizophagus irregularis* DAOM 197198 were more tolerant to low pH than were tomato roots in axenic culture. However, several studies showed that AM fungal colonization was unaffected or was promoted by abiotic stress (Nakatani et al., 2011; Li et al., 2016; Mo et al., 2016). Further analysis is needed because the results will vary depending on the timing of the observation.

Arbuscules are the central sites for nutrient exchange between two symbiotic partners and are therefore considered the core structures of AM symbiosis (Harrison, 2012; Luginbuehl and Oldroyd, 2017). The life span and functional lifetime of arbuscules are only 7.5–8.5 and 2–3 days, respectively (Alexander et al., 1989; Kobae and Hata, 2010), and abiotic stress greatly impedes arbuscule formation (Figure 1; Feng et al., 2020; Liu et al., 2020). A dramatic decrease in arbuscule abundance has been reported under an array of abiotic stresses, such as heavy metal pollution (Zhang et al., 2019), salt stress (Krishnamoorthy et al., 2014; Ye et al., 2019), aluminum toxicity (Göransson et al., 2008), and low pH (Zhu et al., 2007). It is worth noting that the decrease in arbuscule abundance is much greater than that in the abundance of other fungal structures. For instance, arbuscule abundance decreased by 93.27% in contrast to 20.30% for mycorrhizal intensity when the symbiosis was exposed to low pH (Liu et al., 2020). The authors classified arbuscule development into five stages and demonstrated that fewer juvenile arbuscules could develop fully and reach mature status, while mature arbuscules were promoted to become senescent and collapsing in response to low-pH or acidic soil conditions (Feng et al., 2020; Liu et al., 2020). Gutjahr et al. (2012) demonstrated that the half-size ABC transporters STR1 and STR2 were indispensable for arbuscule formation in rice. Intriguingly, arbuscules stopped development but did not collapse in a *str*/*str2* mutant of *Medicago truncatula*, in which the transfer of lipids from host plants to AM fungi was greatly inhibited (Zhang et al., 2010). This different behavior of arbuscules can be explained by the presence of abiotic stresses or not. In str mutant experiment without stress, only the transfer of lipids to AM fungi is inhibited; however, low pH (abiotic stress) not only can inhibit the transfer of lipids to AM fungi but also promote the sporulation (Wang et al., 2017), which requires a huge amount of neutral lipids as storage substance and might drive the collapse of senescent arbuscules to release lipids.

Sporulation determines the spore density in the soil; however, AM fungal sporulation has been less investigated than colonization thus far. Krishnamoorthy et al. (2014) found that the spore density of *Glomus*, *Paraglomus*, *Acaulospora*, *Entrophospora*, *Gigaspora*, and *Scutellospora* exhibited a significantly negative correlation with soil salinity. Yang et al. (2015) reported an overall trend that AM fungal spore density was higher in heavy metal-contaminated soils than in noncontaminated soils. Moreover, elevated temperature was shown to decrease spore density and diameter (Zhang et al., 2016). More intriguingly, a period of drought stress before harvest is frequently practiced to promote sporulation in AM fungal propagation systems (Selvakumar et al., 2018).

## Lipids as Key Nutrients for Arbuscular Mycorrhizal Fungi in Response to Abiotic Stress

Lipids are the most abundant compounds in AM fungi; these lipids mainly include PLs and NLs, but there are also small amounts of other lipids (Bago et al., 2000; Olsson and Johansen, 2000; Wewer et al., 2014). Moreover, AM fungi do not synthesize lipids *de novo* but receive lipids from their hosts (Jiang et al., 2017), indicating the significance of lipids as key nutrients in AM symbiosis.

### Phospholipids in the Membrane (Intraradical and Extraradical Hyphae, and Arbuscules)

The EH and intraradical hyphae (IH), particularly arbuscules, are characterized by large amounts of membrane area, which consists of PLs and phosphate-free lipids called glycolipids (Van Aarle and Olsson, 2003; Wewer et al., 2014). As polar lipid fractions in AM fungi, PLs comprise phosphatidylcholine (PC), phosphatidylethanolamine (PE), phosphatidylinositol, phosphatidylserine, and phosphatidic acid (PA), while glycolipids comprise acylated sterol glucoside, glucosylceramide, and sterol glucoside (Wewer et al., 2014). PLs account for approximately 1–2% of the total lipids in AM fungal biomass, but glycolipids are even more negligible (Jabaji-Hare, 1988; Gaspar et al., 1994; Wewer et al., 2014). PC is the most abundant component of PLs in EH (>60%) and colonized roots (>40%), and furthermore, lyso-PC is considered a signaling molecule during mycorrhization (Drissner et al., 2007; Wewer et al., 2014). However, Gaspar et al. (1997) found that the total contents of PC and PE continuously increased and that the relative contents of PC and PE were dynamic in the roots with AM fungal colonization. Compared with that of PE, the content of PC was greater in the first 3 months, whereas the content of PE was greater in colonized roots after 4 months of AM fungal inoculation (Gaspar et al., 1997). At the molecular level, the expression of monomethyl-PE/dimethyl-PE methyltransferase, which is involved in PC synthesis from PE, and lysophospholipid acyltransferase, which is involved in the interconversion of PC (or PE) and lyso-PC (or lyso-PE), were detected both in colonized roots and in EH (Wewer et al., 2014).

The conversion of PLs to glycolipids is vital in the membranes of both plants and AM during P deprivation to conserve P (Härtel et al., 2000; Wewer et al., 2011, 2014; Pant et al., 2015). Digalactosyldiacylglycerol (DGDG) decreases in parallel with an increase in PC and PE (Wewer et al., 2014). Moreover, relatively low expression of the two *DGDG*s that encode DGDG synthases was observed in colonized roots and noncolonized roots under high-phosphate levels in *Lotus japonicus* due to an increased phosphate supply for PL synthesis (Wewer et al., 2014). Recently, Feng et al. (2020) reported a significant decrease in AM fungal PLs and an accumulation of NLs in colonized roots under low-pH stress (pH 4.5 vs. 6.5), highlighting the interconversion of lipid fractions in AM fungi in response to abiotic stress.

### Neutral Lipids in Storage Structures (Vesicles and Spores)

AM fungi accumulate large amounts of nonpolar storage lipids, i.e., NLs and triacylglycerols (TAGs), mainly in vesicles, extraradical spores, intraradical spores (in some species such as those of *Glomus* and *Rhizophagus*), IH, and EH (Gaspar et al., 1994, 1997; Bago et al., 2000; Wewer et al., 2014). Vesicles are round, elliptical, or irregular in shape and are considered to be the primary storage structures of AM fungi inside roots (Jabaji-Hare et al., 1984; Jabaji-Hare, 1988). Jabaji-Hare (1988) found that NLs in spores and vesicles of several species of *Glomus* and *Rhizophagus* and in spores of *Gigaspora margarita* accounted for 96–98% of total lipids, while polar lipids accounted for 2–4% (Jabaji-Hare, 1988). A continuous decrease in NLs and an increase in PLs were observed in spores during germination (Gaspar et al., 1994). During AM fungal spore germination, lipid synthesis is largely or entirely confined to PL synthesis and, consequently, membrane production (Bago et al., 1999). In AM fungal extraradical structures, spores and hyphae accounted for 90.7 and 9.3% of the total biomass, respectively (Olsson and Johansen, 2000). TAGs are the predominant form of AM fungal lipids; TAGs include 16:0 (palmitic acid) and 16:1ω5 (palmitvaccenic acid) acyl groups (Jabaji-Hare, 1988; Gaspar et al., 1997; Bago et al., 2002; Wewer et al., 2014; Roth and Paszkowski, 2017). Other nonpolar lipids, such as free fatty acids (9–19% of lipids), monoacylglycerols (<9% of lipids), sterol esters (SEs; <7% of lipids), and diacylglycerols (DAGs; <3% of lipids) were also detected in these storage structures (Jabaji-Hare, 1988).

**Figure 2:**
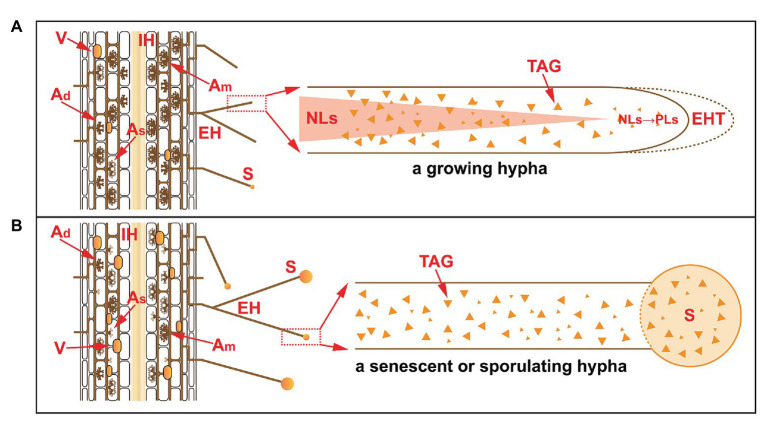
The accumulation of NLs in extraradical hyphae in a growing hypha **(A)** and in a senescent or sporulating hypha **(B)**. Under normal conditions, the life span of arbuscules is 7.5–8.5 days, during which time different stages occur, e.g., developing arbuscules (A_d_), mature arbuscules (A_m_), and senescent arbuscules (A_s_). The PLs in senescent arbuscules are subjected to hydrolysis and converted to NLs for recycling. In the extraradical phase, NLs accumulate but are subsequently consumed. In growing hyphae, NLs are converted to PLs at the front fragment of the hyphae for the synthesis of hyphal tips. In senescent or sporulating hyphae, NLs are transported to developing spores as storage substances. V, vesicles; IH, intraradical hyphae; EH, extraradical hyphae; EHT, extraradical hyphal tip; S, spore; TAG, triacylglycerol; NLs, neutral lipids; PLs, phospholipids.

Previous isotopic labeling experiments and advanced imaging technologies have shown that NLs are synthesized in IH and then transported to EH to sustain extraradical hyphal growth, the formation of new spores, and their subsequent germination (Pfeffer et al., 1999; Bago et al., 2002; Kobae et al., 2014). A continuous mycorrhizal colonization experiment showed a higher accumulation of TAGs than of PLs in EH during the first 3 months after AM fungal inoculation, which accounted for more than 90% of the total lipids in the EH (Gaspar et al., 1997); however, other nonpolar lipids, such as DAGs, free sterols, SEs and PLs, were less abundant (Wewer et al., 2014). In these processes, DAGs serve as the immediate precursor of TAGs, and SEs are involved in the regulation of membrane free sterol homeostasis (Bouvier-Navé et al., 2010). In contrast, the front fragment of growing EH contains fewer NLs than do the rear fragments, probably due to the consumption of NLs for hyphal elongation and sporulation, which implies that continuous NL delivery is probably essential to maintain AM fungal growth (Bago et al., 2002; Kobae et al., 2014). In general, the accumulation of NLs and their conversion to PLs showed different patterns in the growing hyphae and in senescent and sporulating hyphae (Figure 2; Bago et al., 2002), with the latter probably accelerated by increased ABA in response to abiotic stress.

### Potential Conversion of Phospholipids to Neutral Lipids in Arbuscular Mycorrhizal Fungi Under Abiotic Stress

Arbuscules are specialized IHs that account for a large proportion of IHs in terms of membrane surface area in colonized roots. Arbuscule abundance is strongly correlated with the accumulation of PLs across different AM fungal species (Van Aarle and Olsson, 2003; Feng et al., 2020). However, arbuscules are short lived, with a life span of 7.5–8.5 days (Alexander et al., 1989; Kobae and Hata, 2010). This poses a critical issue concerning the fate of PLs after the collapse of arbuscules. A double-staining experiment with vital staining of succinate dehydrogenase and lipid staining (Nile red) revealed that a large number of NLs mainly occurred in senescent arbuscules accompanied by waning vitality and function (Kobae et al., 2014). In line with this result, Feng et al. (2020) observed that there was a distinct negative relationship between arbuscule intactness and NL accumulation, as revealed by fungal cell wall staining (WGA-488) and lipid staining. Furthermore, more dynamic and direct evidence through a live imaging technique indicated that NLs were transported subsequently from IH to EH (Kobae et al., 2014). These observations support that PLs in IH, especially in arbuscules, are hydrolyzed and converted to NLs for recycling. Li et al. (2019a) suggested that a purple acid phosphatase of plant origin was involved in the degradation of arbuscules; however, this was not supported by other experiments (Feng et al., 2020). Therefore, additional work is needed to elucidate the enzymatic mechanism involving the hydrolysis of PLs in collapsing arbuscules.

Arbuscules have a short life span, and furthermore, abiotic stress is capable of accelerating the senescence and collapse of arbuscules. Liu et al. (2020) found that acidic soil resulted in more senescent and collapsed arbuscules in colonized roots. Consistent with this result, Feng et al. (2020) visualized the lipid dynamics in root cells and found that NLs occurred only in senescent and collapsed arbuscule-containing cells. In this scenario, it is highly important to link lipid metabolism in AM fungi with abiotic stress. Molecular analysis indicated that genes encoding a glycerol-3-phosphate acyltransferase (RAM2, involved in lipid biosynthesis) and ATP-binding cassette transporters (STR/STR2, involved in lipid transfer) in colonized roots are greatly inhibited by low pH (Feng et al., 2020). At the physiological level, abiotic stress has been shown to induce ROS generation (Benabdellah et al., 2009) and the visual accumulation of ROS in different structures of AM fungi (Fester and Hause, 2005), leading to membrane lipid peroxidation (malondialdehyde accumulation; González-Guerrero et al., 2007, 2010; Debiane et al., 2011) and a decrease in the accumulation of PLs/PC in AM fungi (Debiane et al., 2011; Calonne et al., 2014; Feng et al., 2020). Evidence of PL dynamics in response to abiotic stress is limited; however, the decrease in PLs is supported by experiments in other organisms. In green algae and *Arabidopsis thaliana*, for example, PLs decrease and are replaced by glycolipids in the membranes, in parallel with the dramatic accumulation in TAGs under P deficiency (Gong et al., 2013; Iwai et al., 2014; Pant et al., 2015). Moreover, abiotic stress impedes P acquisition by AM fungal EH (Wang et al., 2017), which may reduce the P source for PL synthesis in AM fungi. Therefore, abiotic stress can accelerate PL degradation and TAG accumulation, namely, the conversion of PLs to NLs in AM fungi (Calonne et al., 2014; Feng et al., 2020). This conversion can provide at least two benefits: the release of P for recycling under P starvation induced by abiotic stress and an increase in NLs for sporulation in response to abiotic stress.

**Figure 3:**
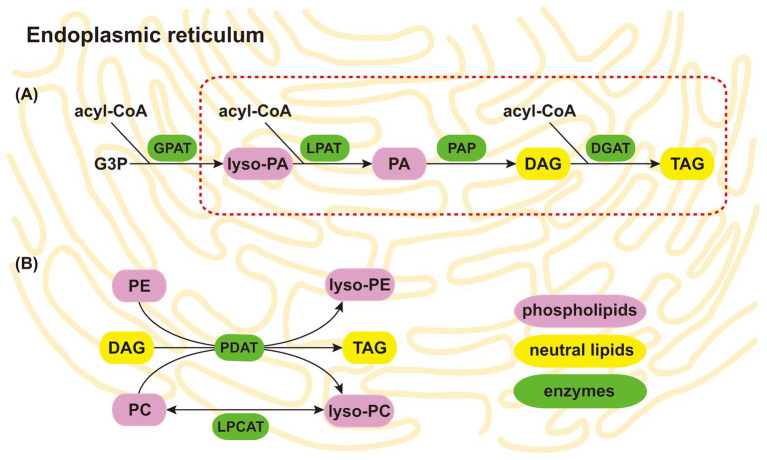
Putative conversion of PLs to NLs in the AM fungal endoplasmic reticulum. Two main pathways include the partial Kennedy pathway, shown as the red dotted line **(A)**, and the acyl editing pathway **(B)**. Considering that PC and PE are the two most abundant species of PLs, the acyl editing pathway is likely the key pathway for converting PLs to NLs in AM fungi. The background represents the AM fungal endoplasmic reticulum. The purple, yellow, and green rectangles represent PLs, NLs, and enzymes, respectively. PA, phosphatidic acid; lyso-PA, lyso-phosphatidic acid; PC, phosphatidylcholine; lyso-PC, lyso-phosphatidylcholine; PE, phosphatidylethanolamine; lyso-PE, lyso-phosphatidylethanolamine; DAG, diacylglycerol; TAG, triacylglycerol; G3P, glycerol-3-phosphate; GPAT, acyl-CoA: glycerol-3-phosphate acyltransferase; LPAT, acyl-CoA:lyso-phosphatidic acid acyltransferase; PAP, phosphatidic acid phosphatase; DGAT, acyl-CoA:diacylglycerol acyltransferase; PDAT, phospholipid:diacylglycerol acyltransferase; LPCAT, acyl-CoA:lyso-phosphatidylcholine acyltransferase; PL, phospholipid; NL, neutral lipid; AM, arbuscular mycorrhizal.

How NLs are produced in collapsed arbuscules remains unknown. It is well acknowledged that a gene encoding cytosolic multidomain fatty acid synthase responsible for the *de novo* synthesis of the bulk of fatty acids is lost in the genome of *Rhizophagus irregularis* (Tisserant et al., 2013; Wewer et al., 2014). Therefore, the transfer of lipids from plant cells to arbuscules is indispensable for AM symbiosis (Jiang et al., 2017). Recently, axenic culture of *R. irregularis* indicated that EH can take up lipids directly from the media and that myristate is the most effective fatty acid to promote hyphal growth, while (S)-12-methyltetradecanoic acid promotes both hyphal growth and sporulation (Kameoka et al., 2019; Sugiura et al., 2019). These results highlight the significance of lipids in AM symbiosis. Under stress conditions, PLs in the hyphal membranes (including arbuscules) undergo hydrolysis and give rise to glycerol-3-phosphate. Given the conversion of PLs to NLs and the accumulation of NLs in AM fungi, as well as the synthesis of NLs in plants and other microbes (Dahlqvist et al., 2000; Oelkers et al., 2000; Arabolaza et al., 2008; Banaś et al., 2013), we speculate that AM fungi employ two main pathways to produce NLs by utilizing PLs (PC, lyso-PC, PE, PA, and lyso-PA) as substrates in the endoplasmic reticulum. In the partial Kennedy pathway, acyl-CoA is utilized for PA synthesis by esterification to the sn-2 positions of lyso-PA by acyl-CoA:lyso-PA acyltransferase. DAGs are subsequently synthesized by the dephosphorylation of PA phosphatase. Finally, DAGs are utilized for TAG synthesis with the participation of acyl-CoA by acyl-CoA:diacylglycerol acyltransferase (DGAT; Figure 3A; Bates and Browse, 2012). In the acyl editing pathway, FAs provided by PC or PE and DAGs are utilized for TAG and lyso-PC synthesis by phospholipid:diacylglycerol acyltransferase (PDAT; Dahlqvist et al., 2000; Oelkers et al., 2000; Bates and Browse, 2012). However, reesterification of lyso-PC by acyl-CoA:lyso-PC acyltransferase generates PC (Figure 3B; Millar et al., 2000; Bates and Browse, 2012). Considering that PC and PE are the two most abundant species of PLs, we infer that the acyl editing pathway is likely the key pathway involved in the conversion of PLs to NLs in AM fungi. The key genes encoding these proteins mentioned above are present in the genome of *R. irregularis* (Tisserant et al., 2013); however, more work is needed to explore this hypothesis. Although few studies have focused on the effects of abiotic stresses on these genes in AM fungi, several of them are upregulated in plants and other microbes, which occurs simultaneously with the accumulation in NLs under abiotic stress (Mus et al., 2013; Zienkiewicz et al., 2016; Yuan et al., 2017; Tan et al., 2018). However, the functional characterization of these genes in AM fungi under various abiotic stresses remains unexplored.

## Abscisic Acid Regulation

As a key “stress hormone,” ABA dramatically accumulates in plants to cope with abiotic stress (Fujii et al., 2009; Kim et al., 2016; Huang et al., 2017b). Apart from its role in plant growth and development, ABA accumulation also directly or indirectly affects plant-associated microbes, such as phytopathogens (Asselbergh et al., 2008) and symbiotic partners, including AM fungi (Liu et al., 2019). Fungal sporulation is a crucial reproductive process and one of the mechanisms by which fungi respond to various adverse conditions, in many cases producing chlamydospores (Spraker et al., 2016; Dijksterhuis 2018). Liu et al. (2019) demonstrated for the first time that exogenous ABA was able to directly improve AM fungal sporulation during both asymbiotic and presymbiotic statuses, highlighting the direct effect of ABA on spores or germ tubes. In AM fungal propagation systems, several weeks of drought stress before harvest is generally believed to induce sporulation (Selvakumar et al., 2016, 2018). ABA accumulation is one of the main responses in plants to drought stress. Therefore, it is likely that there is a positive relationship between ABA and AM fungal sporulation. Nevertheless, it is worth noting that the total spore number decreases under abiotic stress, whereas the sporulation per hypha increases (Debiane et al., 2011), which is supported by the study by Wang et al. (2017). This implies that ABA induces more efficient reproductive growth of AM fungi under abiotic stress. Sporulation involves a large consumption of NLs, which partly originate from the hydrolysis of PLs in arbuscules in response to abiotic stress.

However, in terms of different AM fungal structures, the contents of ABA and ABA-GE as the storage form of ABA in EH were approximately 3-fold and 2.5-fold higher than those in AM fungal spores, respectively (Esch et al., 1994). Remarkably, the content of ABA in AM fungi is more than one order of magnitude higher than that in plant roots (Esch et al., 1994). Pons et al. (2020) provided direct evidence that AM fungi can synthesize cytokinins, auxin, gibberellins, and ethylene but not ABA, which indicates that ABA in AM fungi is derived from plants. Therefore, functional genes responsible for ABA biosynthesis in AM fungi or transporters responsible for transporting ABA from the roots to AM fungi remain unexplored. Additionally, the allocation of ABA among different AM fungal structures (e.g., intraradical vs. extraradical structures) under abiotic stress has not yet been investigated.

To date, no studies have focused on the relationship between ABA accumulation and the conversion of PLs to NLs in AM fungi under abiotic stress. However, the biosynthesis of NLs induced by ABA has been reported in plants, with the plant-AM fungal interaction not considered. In the ABA signaling pathway, the transcription factors *ABSCISIC ACID INSENSITIVE 4* (*ABI4*) and *ABI5* can synergistically trigger stress-induced *DGAT1* expression and TAG accumulation (Yang et al., 2011; Kong et al., 2013). Under nitrogen limitation, *ABI4* promotes TAG accumulation by upregulating the expression of *DGAT1* in *Arabidopsis* (Yang et al., 2011). Recently, Tan et al. (2018) demonstrated that *DGAT1* is critical for freezing tolerance of plants, acting by balancing TAG and PA production in *Arabidopsis*. Additionally, the application of exogenous ABA resulted in an increase in *DGAT1* expression and accumulation of NLs in *Arabidopsis*, which occurred simultaneously with the effects of salt and sorbitol stress (Kong et al., 2013). Various abiotic stresses, including cold, drought, salt, and osmotic stress, increased *PDAT* member expression and TAG accumulation in *Camelina sativa* (Yuan et al., 2017). Multiple analyses and experiments have also shown that *DGAT1*- and *PDAT1*-mediated conversion of membrane lipids into TAGs was enhanced by abiotic stresses in both microalgae and *Arabidopsis* (Yoon et al., 2012; Li et al., 2014; Lee et al., 2019). Based on this evidence, it is likely that ABA is capable of promoting the conversion of PLs to NLs in AM fungi in parallel with the collapse of arbuscules under abiotic stress.

## Conclusions and Perspectives

To date, studies on lipid metabolism in AM fungi in response to abiotic stress are quite rare, given that lipids are an essential component of AM fungi. In this review, we analyze the existing literature, especially studies recently published in this respect, and we speculate that the synthesis and metabolism of lipids may play a key role in AM fungi to acclimate to abiotic stress. In general, under abiotic stress, the expression of AM fungus-specifically induced genes responsible for lipid biosynthesis (*RAM2*) and transfer (*STR/STR2*) from the roots to AM fungi dramatically decreases in parallel with a severe suppression of mycorrhizal colonization and especially a more severe suppression of arbuscule abundance. This may be attributed to the enormous demand for PLs during arbuscule formation. Concomitantly, vesicle formation is suppressed because NLs are required in large amounts for this process. Under abiotic stress conditions, arbuscules become senescent and collapse at a relatively fast rate, which is accompanied by the hydrolysis and conversion of PLs to NLs. Neutral lipids are exported outside through EH to sustain the formation of new spores, which more easily occurs under stress conditions. ABA may act as a signaling molecule during this process, which promotes sporulation in AM fungi.

Most, if not all, relevant studies have concentrated on the plant side with respect to the symbiosis of AM; few studies have paid close attention to the fungal side. This may be due to the impedance of pure cultures of AM fungi. Since some interesting work has recently highlighted pure cultures of AM fungi, especially those concerning the uptake of environmental lipids by fungal hyphae (Sugiura et al., 2019) and the promotive effects of ABA on sporulation (Liu et al., 2019), now is the right time to devote increased effort to strengthen these efforts. For instance, it is essential to clarify whether the enzymes hydrolyzing PLs in senescent arbuscules originate from plants or AM fungi. If they are of plant origin, the elaborate cooperation between two symbiotic partners and signaling is attractive. Second, it is of the utmost importance to explore where ABA in AM fungal structures originates from and what results in the different distribution of ABA in various AM fungal structures, as revealed by Esch et al. (1994). More importantly, whether the process by which NLs produced from the hydrolysis of PLs in collapsed arbuscules are transported to the extraradical spores instead of the intraradical vesicles is related to the different distribution of ABA in intraradical and extraradical fungal structures merits further study. Overall, more studies, especially those on the side of AM fungi, are needed to provide insights into the lipid biology of AM fungi.

## Author Contributions

ZF organized and drafted the manuscript. XL helped in organizing this manuscript. HZ and QY proposed the concept and conceived the structure of this manuscript. QY contributed to the editing of the manuscript. All authors contributed to the article and approved the submitted version.

### Conflict of Interest

The authors declare that the research was conducted in the absence of any commercial or financial relationships that could be construed as a potential conflict of interest.

## Abbreviations

ABA: Abscisic acid
AM: Arbuscular mycorrhiza
DAGs: Diacylglycerols
DGAT: Acyl-CoA:diacylglycerol acyltransferase
DGDG: Digalactosyldiacylglycerol
EH: Extraradical hyphae
G3P: Glycerol-3-phosphate
IH: Intraradical hyphae
NLs: Neutral lipids
PA: Phosphatidic acid
PC: Phosphatidylcholine
PDAT: Phospholipid:diacylglycerol acyltransferase
PE: Phosphatidylethanolamine
PLs: Phospholipids
ROS: Reactive oxygen species
SEs: Sterol esters
TAGs: Triacylglycerols
